# Thinking out of the cranial box in meningitis

**DOI:** 10.1002/agm2.12204

**Published:** 2022-04-01

**Authors:** Sohil Pothiawala, Yuki Tanaka

**Affiliations:** ^1^ Department of Emergency Medicine Woodlands Health Singapore Singapore

## Abstract

Staphylococcus aureus is a leading cause of infective endocarditis. Meningitis is a rare initial presenting feature of *S. aureus* infective endocarditis, especially with the absence of other cardio‐vascular signs. Differentiating patients with uncomplicated *S. aureus* bacteraemia from those with underlying infective endocarditis is often challenging.

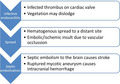

## 1 INTRODUCTION

*Staphylococcus aureus* (*S. aureus*) is a leading cause of bacteremia and infective endocarditis. The mortality rate of *S. aureus* endocarditis is approximately 20%–40%. Mortality is higher in patients who present with neurological complications as an initial manifestation of *S. aureus* endocarditis.^1^ We present a rare case of an 86‐year‐old man who presented to the emergency department (ED) with altered mental state secondary to purulent meningitis as the initial symptom of *S. aureus* endocarditis.

## 2 CASE REPORT

An 86‐year‐old man presented to the ED with a 3‐day history of fever and lethargy. He was noted to be drowsy since the morning of his ED visit. He had a past history of diabetes, hypertension, hyperlipidemia, and hypertrophic cardiomyopathy. He was discharged from the hospital 10 days before his current ED visit, after being admitted for 3 weeks for methicillin‐susceptible Staphylococcus aureus (MSSA) bacteremia from a suspected skin source. During that admission, he was initially treated with intravenous augmentin, and later given a 2‐week course of intravenous cefazolin based on sensitivity of the blood culture as well as consultation with an infectious disease expert. His subsequent blood culture did not grow any bacteria, and his transthoracic echocardiography (TTE) did not show any vegetation and he was discharged well.

**FIGURE 1:**
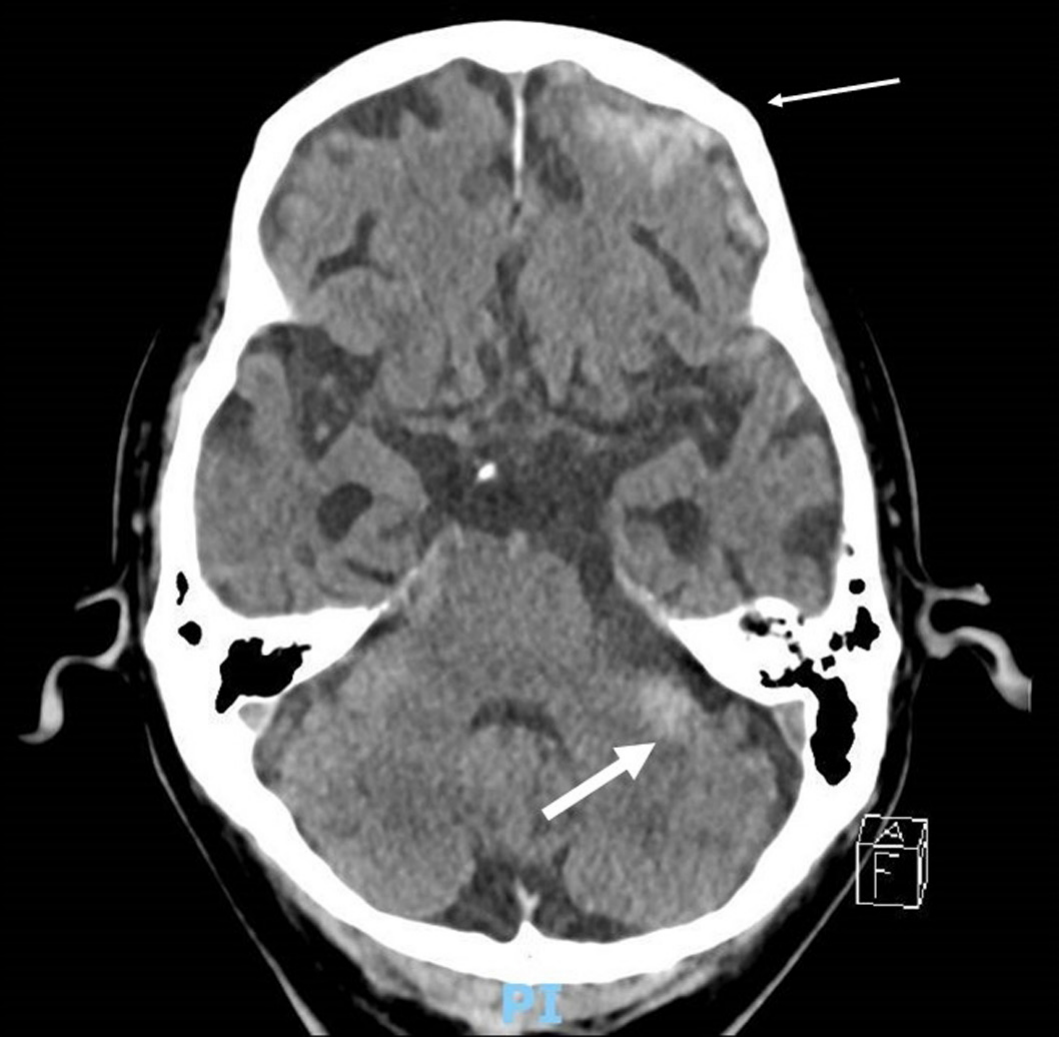
Computerized tomography scan of the brain in the emergency department demonstrating hyperdensities in the subarachnoid spaces along frontal convexity (thin arrow) and a small focus of hyperdensity in the extra‐axial part of left cerebellum (thick arrow), consistent with the diagnosis of purulent leptomeningitis

Upon arrival in the ED, he was febrile at 40°C, heart rate of 111 beats/min, respiratory rate of 32 breaths/min, and blood pressure of 142/68 mm Hg. His GCS was seven (E1V1M5) with reactive pupils and no signs of cellulitis. There were no abnormal findings on examination of the cardiovascular, respiratory, and gastrointestinal system. The breath sounds were normal, and there was no cardiac murmur on auscultation. There was no neck stiffness. Blood tests showed hemoglobin 9.4 g/dl, white cell count of 14.52 × 10^9^/L, platelet count of 61 × 10^9^/L, C‐reactive protein of 321.6 mg/L, bicarbonate 18 mmol/L, and creatinine of 204 μmol/L. severe acute respiratory syndrome‐coronavirus 2 (SARS‐CoV‐2) polymerase chain reaction was negative. A non‐contrast computerized tomography (CT) scan of the brain done in the ED (Figure 1) was reported to have hyperdensities in the subarachnoid spaces along both frontal convexities and a small focus of hyperdensity in the extra‐axial part of the left cerebellum, consistent with the diagnosis of purulent leptomeningitis complicated by subdural empyema. He was commenced on intravenous ceftriaxone, ampicillin, and acyclovir and admitted to the hospital under the internal medicine department.

**FIGURE 2:**
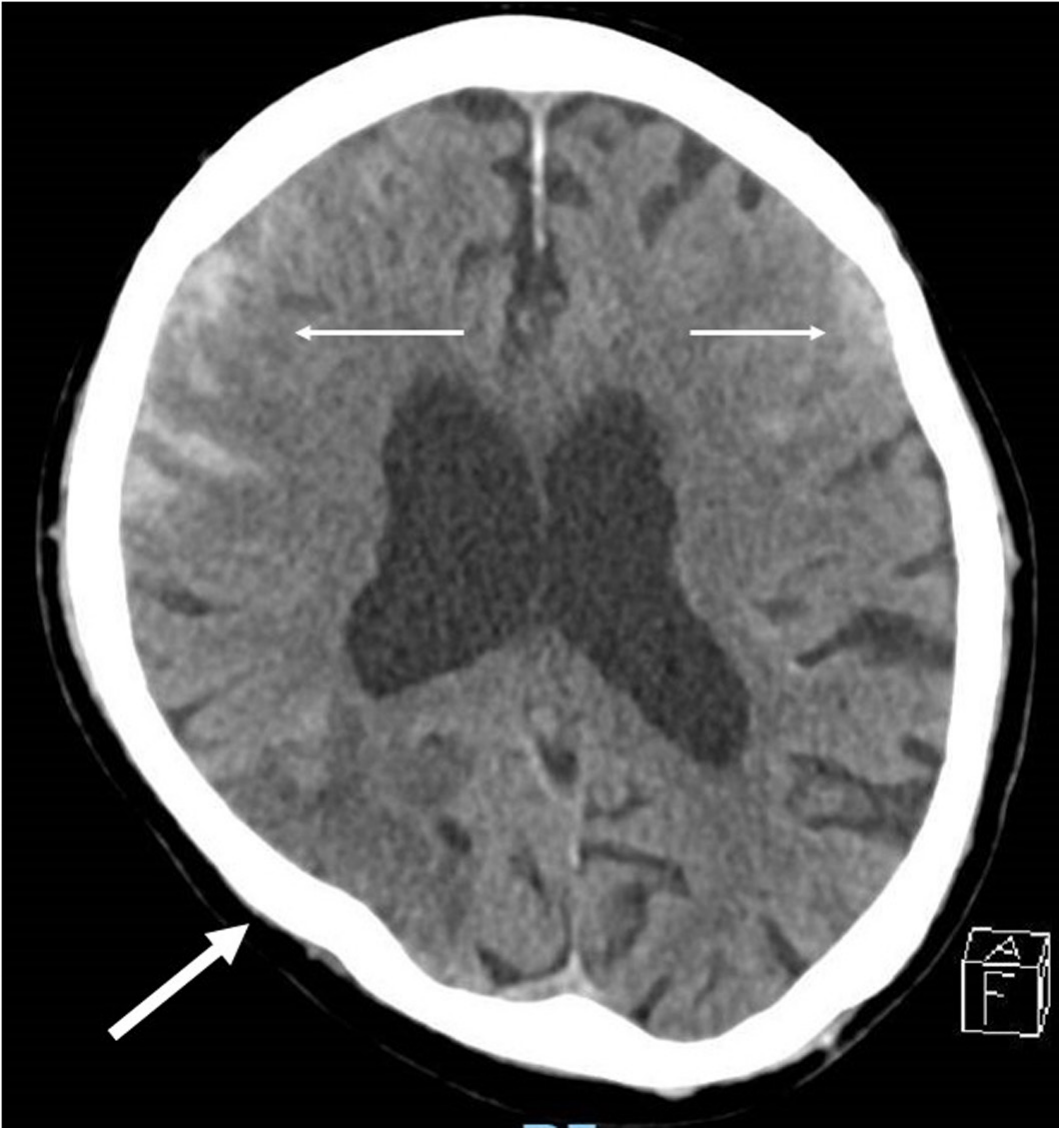
Computerized tomography scan of the brain after 2 days showing subdural/extra‐axial hyperdensity along the left frontal and temporal convexities and right temporal convexity (thin arrow) as well as wedge‐shaped parenchymal hypodensity in the right parieto‐occipital region suggestive of thromboembolic ischemic infarct/septic emboli (thick arrow)

Repeat non‐contrast CT brain after 2 days showed subdural/extra‐axial hyperdensity along the left frontal and temporal convexities, hyperdensities along the right temporal convexity, and a wedge‐shaped parenchymal hypodensity in the right parieto‐occipital region suggestive of thromboembolic ischemic infarct/septic emboli (Figure 2). The development of subarachnoid hemorrhage (SAH) was either secondary to hemorrhagic conversion of the infarct or rupture of a mycotic aneurysm. Blood cultures confirmed MSSA bacteremia and the patient was given intravenous cloxacillin based on culture and sensitivity. Our patient developed a new murmur 5 days later, and a TTE was done which established the diagnosis of *Staphylococcus aureus* infective endocarditis. After discussion with the family, the decision was made for conservative management. Hence, lumbar puncture and magnetic resonance angiography to detect mycotic aneurysm were not done. The patient eventually died.

## 3 DISCUSSION

The neurologic complications of infective endocarditis (IE) usually occur as a result of embolization from the cardiac vegetation.^2^ Some of these manifestations include ischemic stroke (40%–50%), cerebral hemorrhage (12%–30%), and brain abscess (5%).^3^ Nonspecific neurological manifestations include headache, seizures, and toxic non‐focal encephalopathy. Meningitis is a rare presentation of *S. aureus* endocarditis, seen in about 2%–3.5% of the patients,^4^, ^5^ and has a fulminant course and higher mortality. Our patient presented with an altered mental state secondary to leptomeningitis. But he had no other cardiovascular signs suggestive of IE.

Endocarditis is difficult to diagnose initially due to absence of a murmur in up to 30% of the cases at the time of presentation.^3^ Differentiating patients with uncomplicated *S. aureus* bacteremia from those with underlying IE is often challenging. *S. aureus* meningitis usually presents with a primary focus of infection, either pneumonia or endocarditis. Thus, in a case of *S. aureus* infection, endocarditis will precede bacterial meningitis, which occurs later due to septic emboli from the heart valve vegetations, as seen in our case. Hence, the clinical practice guidelines recommend echocardiography in all patients with *S. aureus* bacteremia.^6^

Some patients may also initially present with cerebral hemorrhage, occurring as a result of cerebral vasculitis, hemorrhagic infarction, or ruptured mycotic aneurysm. Intracranial mycotic aneurysms may also be present in < 10% of the cases in patients with *S. aureus* endocarditis,^7^ and are usually secondary to hematogenous spread of septic emboli from the heart. They are located more peripherally compared to berry aneurysm. SAH secondary to ruptured intracranial mycotic aneurysms, is rare, but has a mortality rate of up to 80%.^8^

Multidisciplinary management is the key to reduce mortality in this group of patients. Early diagnosis and initiation of antimicrobial therapy is effective in reducing the neurological complications of IE.^2^ Treatment with antibiotics in these patients with meningitis is recommended for a duration of 4 to 6 weeks, similar to the duration of treatment for IE, which is longer compared to the standard duration of 10 to 14 days for patients with meningitis without endocarditis.^1^, ^9^ Most patients will require valvular surgery after a cerebral embolic event, but the timing of surgery depends on the patient’s comorbid conditions, size of vegetation, risk of recurrent embolization, as well as persistence of vegetations despite antimicrobial therapy.

## 4 CONCLUSION

Physicians should be aware that neurological manifestations may be the initial presenting feature of IE. Considering its rarity and challenges associated with evaluation and diagnosis, early recognition of this condition is crucial to initiate appropriate treatment. Due to the high mortality of purulent meningoencephalitis with *S. aureus* IE, early initiation of antimicrobial therapy and multidisciplinary care, including the appropriate timing of surgery, will aid in reducing the neurological complications of IE and improve outcomes.

## CONFLICT OF INTEREST

All the authors have no conflict of interest.

## AUTHOR CONTRIBUTIONS

S.P. conceived the idea for the manuscript and also contributed to the writing and reviewing of the manuscript. Y.T. contributed to the writing of the manuscript.
